# *Robo2* and *Gen1* Coregulate Ureteric Budding by Activating the MAPK/ERK Signaling Pathway in Mice

**DOI:** 10.3389/fmed.2021.807898

**Published:** 2022-01-05

**Authors:** Yaxin Li, Minghui Yu, Lihong Tan, Shanshan Xue, Xuanjin Du, Xiaohui Wu, Hong Xu, Qian Shen

**Affiliations:** ^1^Department of Nephrology, Shanghai Kidney Development and Pediatric Kidney Disease Research Center, Children's Hospital of Fudan University, Shanghai, China; ^2^State Key Laboratory of Genetic Engineering and National Center for International Research of Development and Disease, Collaborative Innovation Center of Genetics and Development, Institute of Developmental Biology and Molecular Medicine, School of Life Sciences, Fudan University, Shanghai, China

**Keywords:** *Robo2*, *GEN1*, congenital anomalies of the kidney and urinary tract, GDNF/RET, oligo-/polygenic disease

## Abstract

Congenital anomalies of the kidney and urinary tract (CAKUT) are some of the most common developmental defects and have a complicated etiology, indicating an interaction of (epi-) genetic and environmental factors. Single gene mutations and copy number variations (CNVs) do not explain most cases of CAKUT, and simultaneous contributions of more than one gene (di-, oligo-, or polygenic effects; i.e., complex genetics) may lead to the pathogenesis of CAKUT. *Robo2* plays a key role in regulating ureteric bud (UB) formation in the embryo, with mutations leading to supernumerary kidneys. *Gen1* is a candidate gene associated with CAKUT because of its important role in early metanephric development in mice. We established a mouse model with double disruption of *Robo2* and *Gen1* using a *piggyBac* transposon and found that double gene mutation led to significantly increased CAKUT phenotypes in *Robo2*^*PB*/+^*Gen1*^*PB*/+^ mouse offspring, especially a duplicated collecting system. Increased ectopic UB formation was observed in the *Robo2*^*PB*/+^*Gen1*^*PB*/+^ mice during the embryonic period. *Robo2* and *Gen1* exert synergistic effects on mouse kidney development, promoting cell proliferation by activating the GDNF/RET pathway and downstream MAPK/ERK signaling. Our findings provide a disease model for CAKUT as an oligogenic disorder.

## Introduction

Congenital anomalies of the kidney and urinary tract (CAKUT) collectively represent a diverse group of structural malformations that occur during embryonic kidney development, including renal hypoplasia/dysplasia, agenesis, multicystic dysplasia, duplex kidney, hydroureters and ureteropelvic junction obstructions (1). CAKUT is caused by defective kidney development. Mouse kidney development begins at E10.5 with formation of the ureteric bud (UB) from the caudal end of the Wolffian duct (WD), which invades the surrounding metanephric mesenchyme (MM) (2). The UB undergoes T-shaped branching at E11.5 and initiates an arborization program of dichotomous branching events, eventually giving rise to the ureteric tree (3). The MM gives rise to both the cap mesenchyme (nephron progenitor cells), which undergoes a balance of self-renewal and differentiation to sequentially form epithelial nephrons (4, 5), and the stromal elements of the final kidney (6).

The development of the kidney and urinary tract is tightly regulated by multiple genes. However, single gene mutations and copy number variations (CNVs) do not explain the majority of sporadic cases of CAKUT, while complex interactions of multiple genetic and environmental factors may explain a substantial proportion of cases (7). Furthermore, even in mouse models, the phenotypes resulting from genetic deletion vary substantially, suggesting that gene-gene and gene-environment interactions contribute to CAKUT (8). Previous research by our team indicated that a low-protein diet synergizes with *Robo2* mutation to increase the risk of developmental abnormalities in the mouse urinary system (9). Moreover, some mouse polygenic models of CAKUT, such as *Pax2*^+/−^*Pax8*^+/−^ (8), *Hnf1b*^+/−^*Pax2*^+/−^ (9), and *Foxc1*^+/−^*Foxc2*^+/−^ (10) double heterozygous mice, have been established, suggesting that a haploinsufficient genetic combination will result in the CAKUT phenotype. A previous study reported a patient with renal hypodysplasia (RHD) carrying homozygous missense mutations in both *BMP4* (p.N150K) and *DACH1* (p.R684C), which provides a model for RHD as an oligo-/polygenic disorder (11). Therefore, CAKUT is likely associated with multiple gene variants.

Several key regulatory genes involved in normal kidney development in humans and mice, including Roundabout Guidance Receptor 2 (*ROBO2*) and GEN1 Holliday Junction 5′ Flap Endonuclease (*GEN1*), attracted our attention because they play key roles in early urogenital tract development (12–16). ROBO2, as a human CAKUT gene with the OMIM-phenotype number (MIM#610878), a member of the immunoglobulin (Ig) superfamily of cell adhesion molecules (17), binds to its ligand SLIT2 and plays vital roles in maintaining the normal morphogenesis of the kidney. SLIT/ROBO signaling, which is known for its role in axon repulsion, appears to repulse GDNF-expressing cells from the WD, thus causing a physical separation of these two structures in anterior regions (18) and resulting in exposure of the nephrogenic cord to proliferation-inducing signals from which it would normally be shielded by the ureteric mesenchyme. ROBO2 signaling regulates cellular localization by directing the migration of SIX2^+^ cells; thus, the number of SIX2^+^ nephron progenitors (NPs) in the nephrogenic cord increases, resulting in an expansion of the GDNF domain (19). In both *Slit2* and *Robo2* mutants, multiple UBs form anterior to where normal UB outgrowth occurs, and hydroureter and multilobular kidneys are observed (20). In addition, SLIT/ROBO signaling is reportedly involved in regulating branching morphogenesis in the mammary gland and is related to breast cancer development and metastasis (21).

GEN1, a Holliday junction resolvase, is involved in the homologous repair of DNA double strand breaks and in maintaining centrosome integrity (22). *GEN1* may play an important role in the development of the mammary gland, as it is likely involved in the DNA damage response of breast cancer cell lines (23). Our team first discovered that *Gen1* may be a potential candidate gene associated with CAKUT in mice (15). *Gen1* mutation results in kidney abnormalities, including hypoplasia, duplex kidney, hydronephrosis, and the vesicoureteral reflux (VUR), in mice (15). During early kidney development, *Gen1* mutation impairs GDNF expression, resulting in defective UB branching during early kidney development. The expression level of the cap mesenchyme marker SIX2 was also decreased in *Gen1* mutant kidney primordia, indicating that the smaller cap mesenchyme impaired metanephros differentiation and led to kidney hypoplasia in *Gen1* mutants (16).

Both *Robo2* and *Gen1* are involved in kidney development by regulating the GDNF/RET pathway, thus affecting ureteric budding and the development of the nephron progenitors. Together, these observations prompted us to assess whether *Robo2* and *Gen1* cooperate in a common signaling pathway during mouse kidney development *in vivo*. Therefore, in the present study, we established a mouse model with *Robo2* and *Gen1* double mutations to observe postnatal urinary malformations and early-stage kidney developmental abnormalities and investigate whether double gene mutations increase the incidence of CAKUT and the underlying mechanism.

## Materials and Methods

### Mice

*Gen1*^*PB*/+^ mice were obtained by using PB transposon-based insertional mutagenesis targeted to intron 2 of *Gen1* (Chr: 12.11268138, Ensembl: ENSMUSG00000051235). *Robo2*^*PB*/+^ mice were also obtained through PB transposon-based insertional mutagenesis targeted to intron 1 of the *Robo2* gene (Chr: 16.74379623, Ensembl: ENSMUSG00000052516). *Gen1*^*PB*/+^ and *Robo2*^*PB*/+^ mice were mated with *Hoxb7*/myr-Venus mice to obtain *Gen1* and *Robo2* PB insertion mice specifically expressing fluorescent protein in the UB epithelium (*Gen1*^*PB*/+^*;Hoxb7*/myr-Venus abbreviated as *Gen1*^*PB*/+^*;Hoxb7* and *Robo2*^*PB*/+^*; Hoxb7*/myr-Venus abbreviated as *Robo2*^*PB*/+^*; Hoxb7*). *Gen1*^*PB*/+^*;Hoxb7* mice were mated with *Robo2*^*PB*/+^*;Hoxb7* mice to obtain *Gen1*^*PB*/+^ *Robo2*^*PB*/+^*;Hoxb7, Gen1*^*PB*/+^*;Hoxb7, Robo2*^*PB*/+^*;Hoxb7* and WT mice. We observed the phenotypes of the mice urinary system by selecting the same nest of different genotypes. All mice used in the experiments were specific pathogen-free (SPF) and were maintained at 18–22°C and 50–60% humidity on a 12 h day/night cycle. Animals were maintained and managed according to the animal welfare and usage management regulations of the School of Life Sciences of Fudan University [Protocol Approval No. SYXK (hu) 2020–0011]. All mouse strains were maintained on an FVB/N background.

### Histological Analyses

Hematoxylin and eosin (H&E) staining was carried out on 7 μm paraffin sections, and performed according to the standard protocols described in the literature (24).

### Quantitative Real-Time PCR

Total RNA was extracted from E12.5 using TRIzol (Life Technologies, United States). Total RNA was extracted from kidney primordia of E10.5 embryos using a RNeasy mini kit (Qiagen, Germany). The purity of the RNA was confirmed by measuring the ratio of optical densities at 260 nm/280 nm. Aliquots of total RNA (1.0 μg each) from each sample were reverse transcribed into cDNAs according to the instructions of the PrimeScript^®^ RT Reagent Kit (TaKaRa, China). Amplification was performed using AceQ qPCR SYBR Green Master Mix (Vazyme, China) and a real-time qPCR system (Agilent Mx3000P, United States). *Gapdh* was used as the internal control. All primers were obtained from the NCBI GenBank database and were synthesized by Genewiz (Shanghai, China). The primers used for RT-PCR were as follows (5′-3′): *Gen1*-F: 5′-GCCTGGAGTTGGAAAGGAACAAG-3′, *Gen1*-R: 5′-GGAACACACAGAGCAGTGAACCAC-3′; *Robo2*-F: 5′-GCGGATCTTTATTCTTTTTGCG-3′, *Robo2*-R: 5′-TCCTTTTTCCAGTAGATGGTTG-3′; *Gdnf*-F: 5′- GAACCAAGCCAGTGTATCTCCT-3′, *Gdnf*-R: 5′-ATCGTCTCTGCCTTTGTCCTC-3′; *Ret*-F: 5′-TGGCACACCTCTGCTCTATG-3′, *Ret*-R: 5′-GATGCGGATCCAGTCATTCT-3′; *Grem1*-F: 5′-CCTTTCAGTCTTGCTCCTTCTGC-3′, *Grem1*-R: 5′-TTCTTCTTGGTGGGTGGCTGTAGC-3′; *Bmp4*-F: 5′-GCAAGTTTGTTCAAGATTGGCTCC-3′, *Bmp4*-R: 5′-CCATCAGCATTCGGTTACCAGG-3′; *Bmp2*-F: 5′-CACACAGGGACACACCAACC-3′, *Bmp2*-R: 5′-CAAAGACCTGCTAATCCTCAC-3′; *Six2*-F: 5′- AGTCCGACGTGATGTGAAC-3′, *Six2*-R:5′-AGAGAAATGAGAATTCAGGTGC-3′; *Foxc1*-F: 5′-GCCAAATGGAATGGAACCCC-3′, *Foxc1*-R: 5′-CGCTGGTGTGAGGAATCTTCTC-3′; *Slit2*-F: 5′-CCTGTGATGATGGAAATGATGAC-3′, *Slit2*-R: 5′-CCACTGTATCCAAGCAGGT-3′; and *Gapdh*-F: 5′-TGTTCCTACCCCCAATGTGTCC-3′, *Gapdh*-R: 5′-GGAGTTGCTGTTGAAGTCGCAG-3′. The relative gene expression levels were calculated using the 2^−ΔΔCT^ method. Each group contained at least three samples, and each sample was assayed in triplicate.

### Phenotype Analysis

After the newborn mice in each group were anesthetized with CO_2_, the abdomen was exposed along the midline incision of the anterior abdominal wall. Liver and intestinal tissues were removed to make sure that the urinary systems of the mice were fully exposed. The sex of the mice was determined under a fluorescence stereoscope, and the location, morphology, number of kidneys, ureters and bladders in the newborn pups from each group were observed. Pregnant mice were anesthetized at E11, E11.5 and E12.5 using CO_2_. The abdomen of the mice was fully exposed, and the uteri were separated. The placenta and fetal membranes of the mice were removed under a stereoscope. The mouse embryos were flattened, limbs, intestines and liver were removed, and the bean-shaped tissue near the spinal column on the posterior abdominal wall was identified as early embryonic kidney tissue. The location and number of UBs were observed. The images were recorded under a microscope (Leica, Germany).

### Whole Mount Immunofluorescence Staining

Embryos were dissected at E11.5 and fixed in 4% paraformaldehyde solution (PFA) (pH 7.4) at 4°C overnight. After the tissues were soaked in PBS/1% Triton X-100 for 20 min and rinsed with PBS several times, they were blocked with PBS (0.3% Triton X-100, 5% normal donkey serum and 1x PBS) overnight at 4°C. The tissues were incubated overnight at 4°C with primary antibodies against phospho-ERK (Cell Signaling Technology, 4,370; 1:100), phospho-AKT (Cell Signaling Technology, 8,272; 1:100), and phospho-PLCγ (Cell Signaling Technology, 8,713; 1:100). The tissues were washed 3 times with PBS for 30 min each, incubated with Cy5-conjugated donkey anti-rabbit secondary antibody (Jackson, 1:500) overnight at 4°C and washed several times with PBS. Images of stained tissue were captured using a Zeiss-LSM880 with Airyscan confocal laser scanning microscope and ZEN ImageJ software (Zeiss, Germany). The details about how the measurements were conducted on the confocal images are described in the literature (25). ImageJ was used to quantify the amount of fluorescence as mean gray value of pERK, pAKT, PLCγ and compare the results among four groups. ImageJ was also used to determine the number of stained cells by phosphorylation of histone H3 (PHH3) to compare the results among these groups.

### Statistics

All data were processed using STATA 16.0 statistical software. The data are presented as the means ± SEM. The differences between two groups were determined with an unpaired *t*-test or Fisher's exact test. The significance level was set to *P* < 0.05.

## Results

### Decreased *Robo2* and *Gen1* Expression in the *Robo2^PB/+^Gen1^PB/+^* Mutant Mice

**Figure 1:**
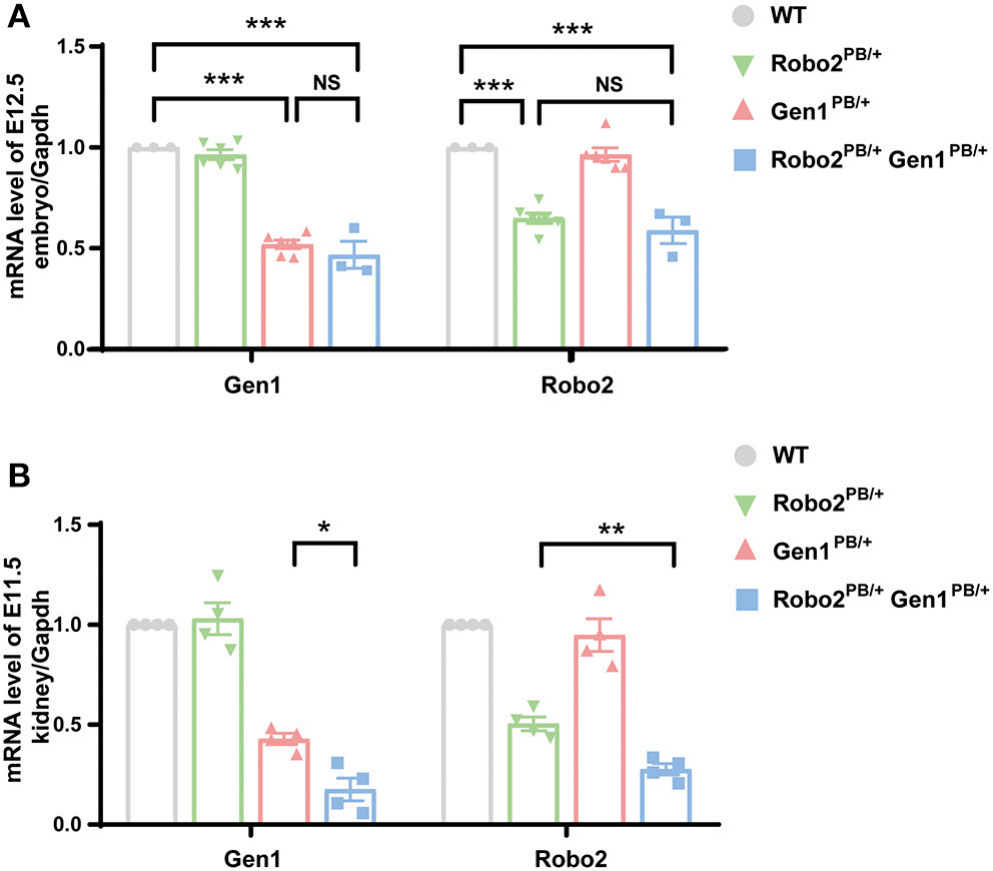
Expression of *Robo2* and *Gen1* in the WT, *Robo2*^*PB*/+^, *Gen1*^*PB*/+^ and *Robo2*^*PB*/+^*Gen1*^*PB*/+^ mice. **(A)** Expression levels of *Gen1* and *Robo2* in E12.5 whole WT, *Robo2*^*PB*/+^*, Gen1*^*PB*/+^, and *Robo2*^*PB*/+^*Gen1*^*PB*/+^ mouse embryos. *N* = 3 WT, *Robo2*^*PB*/+^ and *Robo2*^*PB*/+^*Gen1*^*PB*/+^ embryos each; N = 5 *Gen1*^*PB*/+^ embryos. **(B)** Expression levels of *Gen1* and *Robo2* in kidneys from E11.5 WT, *Robo2*^*PB*/+^*, Gen1*^*PB*/+^ and *Robo2*^*PB*/+^*Gen1*^*PB*/+^ mice. N = 4 mice each in the four groups. Data are presented as the means ± SEM. NS, nonsignificant; *P < 0.05; **P < 0.001; ***P < 0.0001.

The insertion of a *piggyBac* (PB) transposon into the second intron of the mouse *Gen1* gene and the first intron of the *Robo2* gene significantly disrupted their expression. We analyzed the expression of *Robo2* and *Gen1* in the control and mutant mice on E12.5 using RT-PCR. In the whole embryos of the *Robo2*^*PB*/+^*Gen1*^*PB*/+^ mutant mice, *Robo2* expression significantly decreased by 41% compared with that in the WT mice (*P* < 0.0001), but *Robo2* expression was not different from that in the *Robo2*^*PB*/+^ mutant mice. In the whole embryos of the *Robo2*^*PB*/+^*Gen1*^*PB*/+^ mutant mice, the expression level of *Gen1* was significantly decreased by 53% compared with that in the WT mice (*P* < 0.0001), but its expression was not different from that in *Gen1*^*PB*/+^ mutant mice (Figure 1A).

We also analyzed the expression of *Robo2* and *Gen1* in the embryonic kidney tissue on E11.5. *Robo2* was expressed at lower levels in embryonic kidney tissue from the *Robo2*^*PB*/+^*Gen1*^*PB*/+^ mutant mice than in the WT mice (*P* < 0.0001) and the *Robo2*^*PB*/+^ mutant mice (*P* = 0.002). Lower expression of *Gen1* was detected in the embryonic kidney tissue of the *Robo2*^*PB*/+^*Gen1*^*PB*/+^ mutant mice than in the WT mice (*P* = 0.004) and the *Gen1*^*PB*/+^ mutant mice (*P* = 0.03) (Figure 1B).

### Increased Presentation of CAKUT Phenotypes in the Neonatal *Robo2^PB/+^Gen1^PB/+^* Mutants

**Figure 2:**
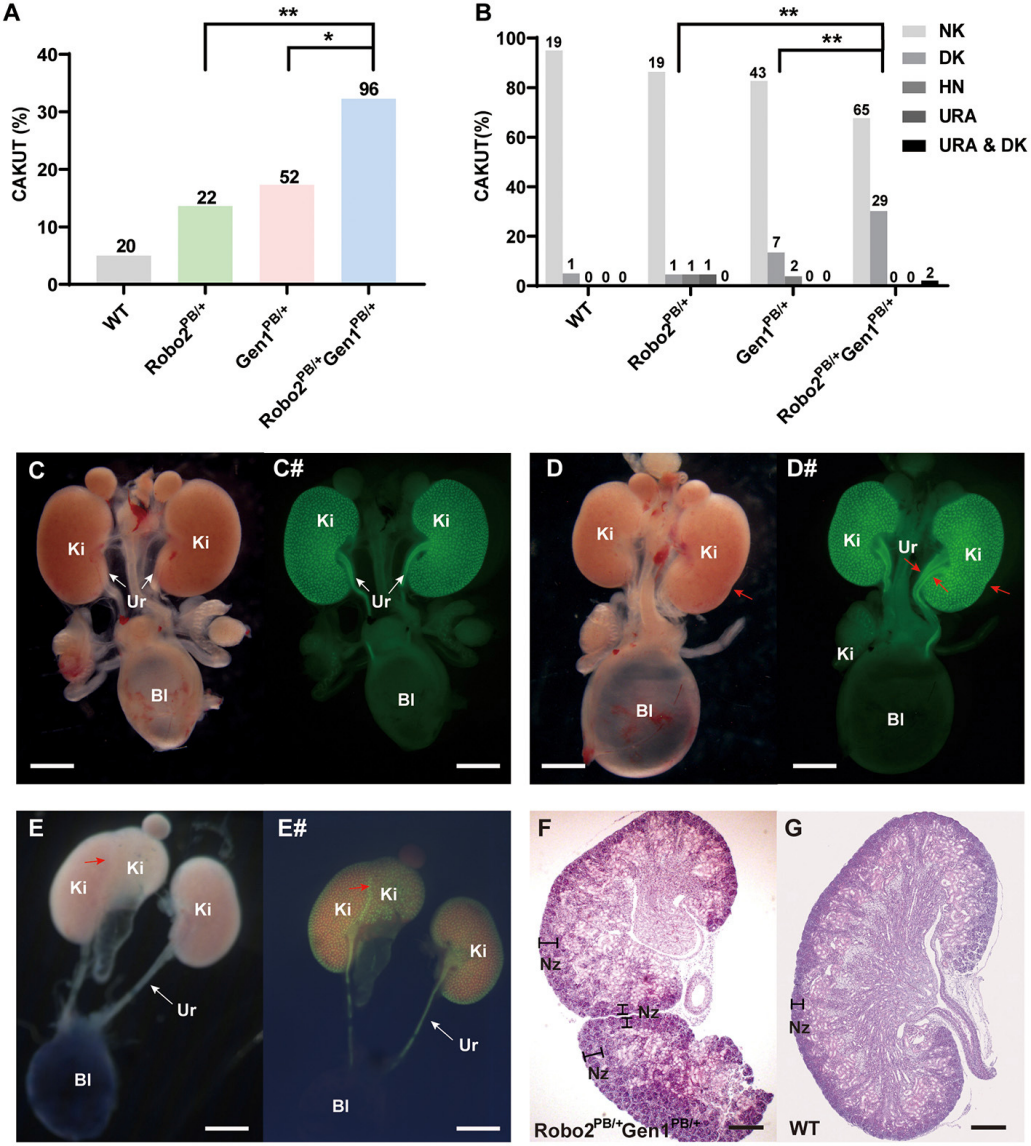
Analysis of newborn mouse phenotypes in the four groups at P0.5. **(A)** Summary of the kidney symptoms in the four groups. **(B)** Percentages of newborn mice with a number of normal kidneys (NK), duplex kidneys (DK), hydronephrosis (HN), unilateral renal agenesis (URA) and hydronephrosis complicated with duplex kidneys (HN & DK). The number n represents the number of cases. **(C–E)** Representative images of the normal kidney **(C)**, duplex kidney **(D)**, and hydronephrosis complicated with duplex kidney **(E)**. **(C#–E#)** Representative images visualized by staining for *Hoxb7* expression in normal kidneys **(C#)**, duplex kidneys **(D#)**, and hydronephrosis complicated with duplex kidneys **(E#)**. Red arrowheads in **(D–E#)** indicate abnormal nephrogenic zones of the duplex kidney and double ureters. White arrows indicate the normal ureter. **(F,G)** Histological sections of P0.5 *Robo2*^*PB*/+^*Gen1*^*PB*/+^ **(F)** and WT kidneys **(G)** stained with hematoxylin and eosin. The region where nephrogenesis occurs (nephrogenic zone) indicated by black lines is restricted to the periphery in the WT kidney but extends toward the inner cortex of the *Robo2*^*PB*/+^*Gen1*^*PB*/+^ kidney. Scale bars, 2 mm in **(C–E#)**; 200 μm in **(F,G)**. NK, normal kidney; DK, duplex kidney; URA, unilateral renal agenesis; HN, hydronephrosis; Bl, bladder; Ki, kidney; Ur, ureter; Nz, nephrogenic zone; NS, non-significant; *P < 0.05; **P < 0.001.

Ninety-six newborn *Robo2*^*PB*/+^*Gen1*^*PB*/+^ mice were grossly dissected for a comprehensive analysis of the urinary system. The number of mice used in each group was listed in a table (Supplementary Table 1). The incidence of CAKUT in the neonatal period was 32.3%, which was higher than that in the *Robo2*^*PB*/+^ group [32.3% (31/96) vs. 13.6% (3/22), *P* = 0.01] and in the *Gen1*^*PB*/+^ group [32.3% (31/96) vs. 17.3% (9/52), *P* = 0.038] (Figure 2A). Among these CAKUT phenotypes, the incidence of duplex kidney was increased in *Robo2*^*PB*/+^*Gen1*^*PB*/+^ newborn mice compared with the *Robo2*^*PB*/+^ group [30.2% (29/96) vs. 4.5% (1/22), *P* = 0.017] and the *Gen1*^*PB*/+^ group [30.2% (29/96) vs. 13.5% (7/52), *P* = 0.045] (Figure 2B). Five percent (1/20) of the WT newborn mice showed duplex kidneys. The incidence of duplex kidney, renal dysplasia (Supplementary Figure 1A) and hydronephrosis (Supplementary Figure 1B) was 4.5% (1/22) in the *Robo2*^*PB*/+^ neonatal mice. Thirteen percent (7/52) of the *Gen1*^*PB*/+^ newborn mice showed duplex kidneys, and 3.8% (2/52) of the mice showed hydronephrosis (Figure 2B). The CAKUT phenotype (Figures 2C–E#) of the *Robo2*^*PB*/+^*Gen1*^*PB*/+^ mice included isolated duplex kidneys (30.2%, 29/96, Figures 2D,D#) and hydronephrosis complicated with duplex kidneys [2.1%, (2/96), Figures 2E,E#], with isolated duplex kidneys being the most common phenotype. No sex or site preference was observed.

In the duplex kidney, the nephrogenic zone where new nephrons are being generated, which is normally restricted to the periphery of the developing kidney (Figure 2F), extended toward the inner cortex of the mutant kidneys (Figure 2G).

We also examined urinary tract defects in 36 newborn *Robo2*^*PB*/+^*Gen1*^*PB*/+^ mice and confirmed VUR in nine mutant mice. Three refluxes were observed in all 22 *Gen1*^*PB*/+^ newborn mice, and only one of the 10 *Robo2*^*PB*/+^ mutant mice had confirmed VUR. In the mutant mice with VUR, we observed methylene blue reflux to the renal pelvis and no significant renal pelvic or ureteral dilatation. However, no significant difference in the proportion of VUR was observed among these three groups (Supplementary Figure 1).

### Duplex Kidneys Arise From Defective Kidney Induction in the *Robo2^PB/+^Gen1^PB/+^* Mutants

**Figure 3:**
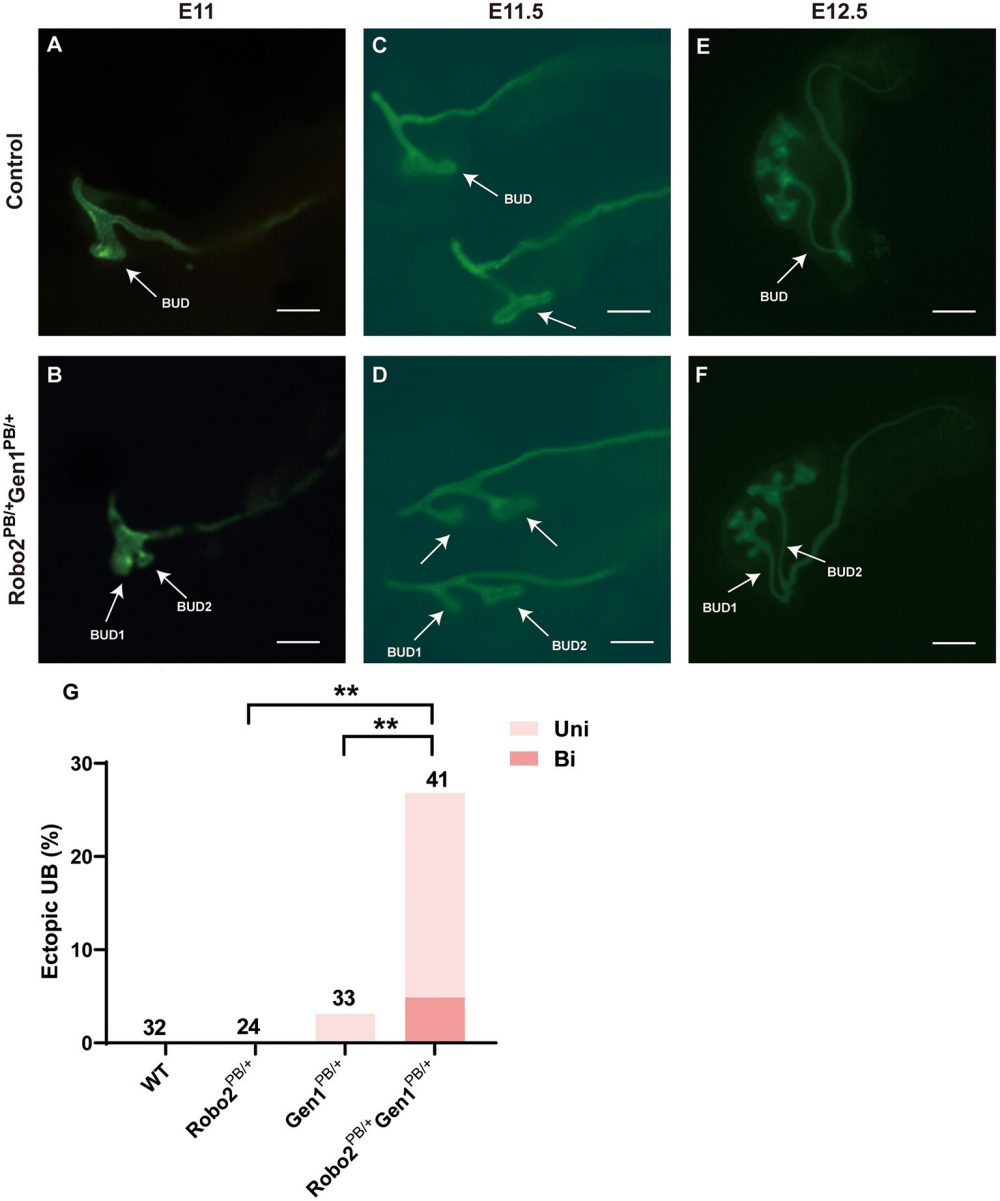
Comparison of ureteric budding in the four groups. **(A–F)** Staining of whole mounts of the *Robo2*^*PB*/+^*Gen1*^*PB*/+^ mutants at E11 **(A)**, E11.5 **(C)**, and E12.5 **(E)** visualized using *Hoxb7*/myr-Venus expression shows abnormal ectopic ureteric budding compared with the single normal UB observed in the WT mice **(B,D,F)**. **(G)** Summary of the ectopic budding incidence in the WT, *Robo2*^*PB*/+^, *Gen1*^*PB*/+^, and *Robo2*^*PB*/+^*Gen1*^*PB*/+^ kidneys. The ectopic budding incidence is shown as the number of kidneys with ectopic budding or branching/total number of kidneys examined. Uni, Unilateral; Bi, Bilateral. Scale bars represent 100 μm in A-D. Scale bars represent 200 μm in E-F. NS, non-significant; **P < 0.001.

Excessive UB formation was reported to cause duplex kidneys. *Hoxb7*/myr-Venus specifically expresses a fluorescent protein in the UB epithelium. We introduced *Robo2* and *Gen1* mutations in *Hoxb7* transgenic mice by breeding to visualize the morphology of the UB in mutant mice, and the UB and nephric ducts were positive for green fluorescence. From E11–E12.5, when the UB invaded the MM, abnormal ectopic budding from the WD was observed (Figure 3, compare **D–F** with **A–C**). The *Robo2*^*PB*/+^ and *Gen1*^*PB*/+^ mutant embryos had a single UB at the typical T-stage (E11.5), whereas the *Robo2*^*PB*/+^*Gen1*^*PB*/+^ mutants frequently showed ectopic budding that had already branched from the main UB. No ectopic budding was observed in any of the 32 WT mice or in any of the 24 *Robo2*^*PB*/+^ embryonic control mice. Three percent (1/33) of the *Gen1*^*PB*/+^ mutant kidneys showed ectopic budding compared with 26.8% (11/41) of the *Robo2*^*PB*/+^*Gen1*^*PB*/+^ kidneys (0/32 vs. 11/41, *P* = 0.002; 0/24 vs. 11/41, *P* = 0.005; 1/33 vs. 11/41, *P* = 0.005). The proportion of unilateral ectopic budding in *Gen1*^*PB*/+^ kidneys was 1/33 (3.0%) compared with 22.0% (9/41) in *Robo2*^*PB*/+^*Gen1*^*PB*/+^ kidneys (0/32 vs. 9/41, *P* = 0.001; 0/24 vs. 9/41, *P* = 0.003; 1/33 vs. 9/41, *P* = 0.018) (Figure 3G), suggesting that *Robo2* and *Gen1* may synergistically regulate ureteric budding to prevent duplex kidney formation.

### GDNF/RET Signaling Is Enhanced in the *Robo2^PB/+^Gen1^PB/+^* Mutants

**Figure 4:**
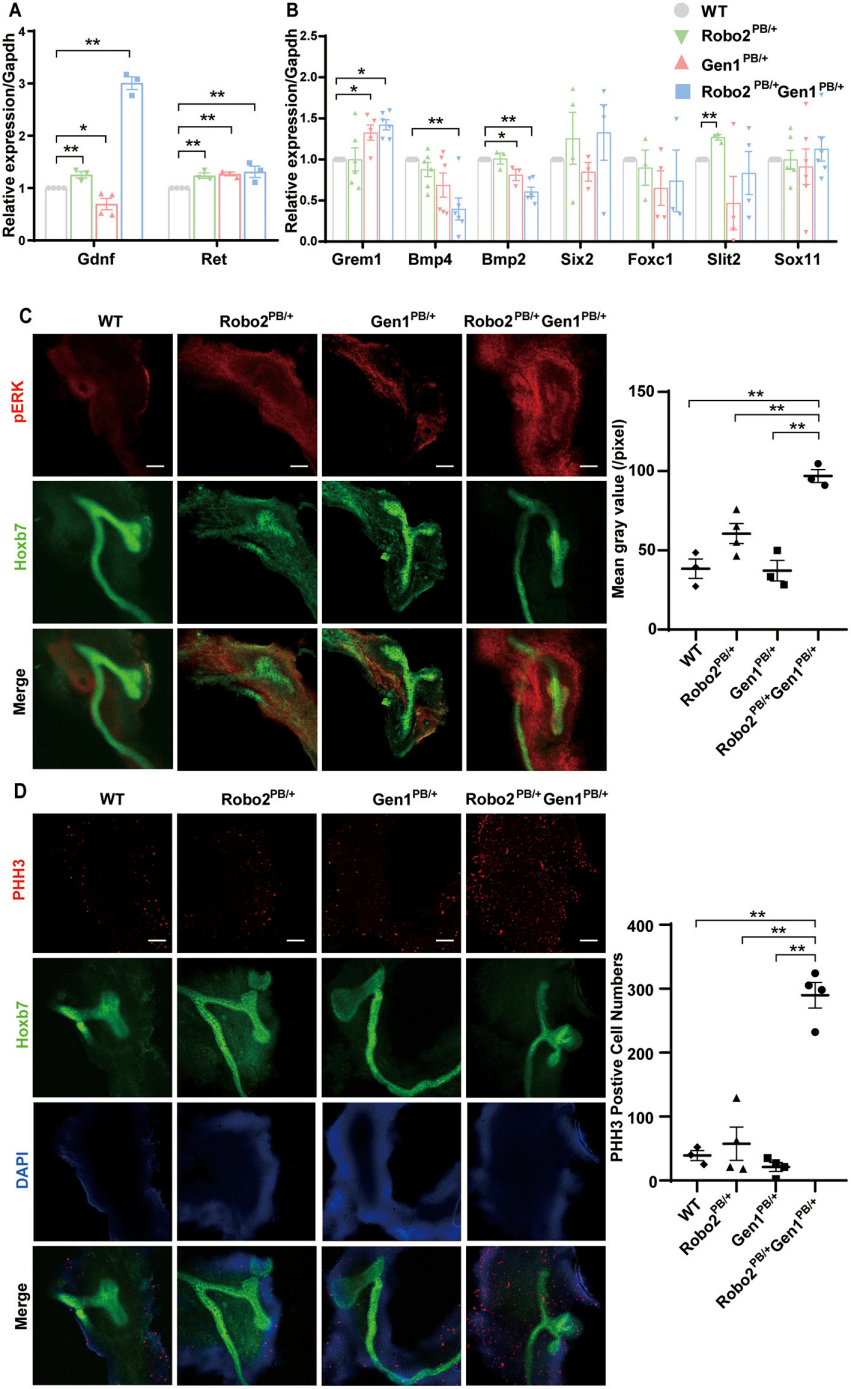
GDNF/RET signaling is increased in the *Robo2*^*PB*/+^*Gen1*^*PB*/+^ mutants during metanephric development. **(A)** Changes in *Gdnf* and *Ret* expression in kidneys from the E11.5 WT, *Robo2*^*PB*/+^*, Gen1*^*PB*/+^ and *Robo2*^*PB*/+^*Gen1*^*PB*/+^ mice, *N* = 3. **(B)** Changes in the expression of key genes involved in metanephric development at E11.5, *N* = 3. **(C)** Immunofluorescence staining with antibodies against pERK in the kidneys of the WT, *Robo2*^*PB*/+^, *Gen1*^*PB*/+^, *Robo2*^*PB*/+^*Gen1*^*PB*/+^ mice at E11.5. *N* = 3, original magnification × 10. **(D)** Immunofluorescence staining with antibodies against PHH3 in the kidneys of the WT, *Robo2*^*PB*/+^, *Gen1*^*PB*/+^, *Robo2*^*PB*/+^*Gen1*^*PB*/+^ mice at E11.5. *N* = 4, original magnification × 10. Scale bars represent 100 μm in **(C,D)**. *P < 0.05; **P < 0.001.

The *Robo2*^*PB*/+^*Gen1*^*PB*/+^ mutants displayed several phenotypes that may be caused by increased GDNF/RET signaling. We examined the expression of *Gdnf* and *Ret* in embryonic kidney tissues at E11.5 using RT-PCR to determine whether RET signaling is increased in the *Robo2*^*PB*/+^*Gen1*^*PB*/+^ mutants. Compared with the WT mice, *Gdnf* expression was decreased in the *Gen1*^*PB*/+^ mutant mice (*P* = 0.03), significantly increased both in the *Robo2*^*PB*/+^ mutant mice (*P* < 0.001), and in the *Robo2*^*PB*/+^*Gen1*^*PB*/+^ mutant mice (*P* < 0.001). *Ret* expression was significantly increased in the *Robo2*^*PB*/+^, *Gen1*^*PB*/+^ and *Robo2*^*PB*/+^*Gen1*^*PB*/+^ (*P* < 0.001) mutant mice (Figure 4A).

Then, we detected the expression of genes involved in regulating the GDNF/RET signaling axis and causing duplex kidney: *Gremlin* (*Grem1*), *Bmp4, Bmp2, Six2, Foxc1, Slit2* and *Sox11*. Among them, *Grem1* expression was increased in the *Gen1*^*PB*/+^ mutant mice (*P* = 0.008) but not in the *Robo2*^*PB*/+^ mutant mice. *Grem1* expression was increased in the *Robo2*^*PB*/+^*Gen1*^*PB*/+^ mutant mice (*P* = 0.04), while both *Bmp2* and *Bmp4* expression levels were markedly decreased in the *Robo2*^*PB*/+^*Gen1*^*PB*/+^ mutant mice (*P* < 0.001). *Bmp2* was also reduced in the *Gen1*^*PB*/+^ mutant mice (*P* = 0.04). *Slit2* expression was significantly increased in the *Robo2*^*PB*/+^ mutant mice compared with the WT mice (*P* < 0.001), but not in the *Gen1*^*PB*/+^ and *Robo2*^*PB*/+^*Gen1*^*PB*/+^ mutant mice. *Six2, Foxc1, Slit2*, and *Sox11* expression levels were not changed in the four groups (Figure 4B).

We examined the phosphorylation of a downstream effector, ERK. pERK has a wide range of both cytosolic and nuclear targets and functions, and its level reflects MAPK signaling downstream of RET. We also examined the phosphorylation of two other downstream effectors, AKT and PLCγ, which are both involved in RET intracellular signaling cascades. The pERK level was significantly increased in the UB and its surrounding nephrogenic cord cells in the E11.5 *Robo2*^*PB*/+^*Gen1*^*PB*/+^ kidneys compared with the WT (*P* = 0.0014), *Robo2*^*PB*/+^ (*P* = 0.0069), and *Gen1*^*PB*/+^ kidneys (*P* = 0.0015) (Figure 4C). However, we were unable to detect alterations in pAKT and pPLCγ levels in E11.5 kidneys (Supplementary Figure 2). The MAPK pathway, which involves the ERK1/2 cascade, is activated by various stimuli and contributes to the regulation of proliferation, differentiation, and cell survival.

We detected the expression of the RET transcription factor Etv5 to determine whether MAPK/ERK signaling was upregulated in *Robo2*^*PB*/+^*Gen1*^*PB*/+^ mutants. We found increased expression of Etv5 at E12.5, when the ureteric buds were undergoing repeated branching events (Supplementary Figure 3). We also determined the level of PHH3, which is an indicator of mitosis. Importantly, in the E11.5 *Robo2*^*PB*/+^*Gen1*^*PB*/+^ kidney sections, PHH3 levels were significantly increased compared with the WT (P < 0.001), *Robo2*^*PB*/+^ (P = 0.0005), and *Gen1*^*PB*/+^ groups (P < 0.001) (Figure 4D).

## Discussion

In this study, we showed that double heterozygous mutation for *Robo2* and *Gen1* led to significantly increased CAKUT phenotypes, particularly a duplicated collecting system, indicating a genetic interaction between the two genes. Furthermore, compound heterozygosity results in more ectopic UBs and cell proliferation through the activation of the GDNF/RET pathway and downstream MAPK/ERK cascade.

In our study, the *Robo2*^*PB*/+^*Gen1*^*PB*/+^ mice exhibited an aggravation of the CAKUT phenotype, and the incidence of CAKUT was higher than that in the *Robo2*^*PB*/+^ and *Gen1*^*PB*/+^ mice. In addition, the phenotypic distribution of the *Robo2*^*PB*/+^*Gen1*^*PB*/+^ mice was relatively concentrated, and duplex kidneys were mainly observed. The proportion of ectopic buds detected in the embryonic stage of mice with double gene mutations was significantly increased. The expression of *Robo2* and *Gen1* in the embryonic kidney of *Robo2*^*PB*/+^*Gen1*^*PB*/+^ mutant mice, but not in general in whole embryos, was lower than that in *Robo2*^*PB*/+^ and *Gen1*^*PB*/+^ mice, suggesting that *Gen1* and *Robo2* may have interaction and *Gen1* expression seems to depend on *Robo2* expression during kidney development. GDNF expression was decreased at E11.5 in the *Gen1*^*PB*/+^ mutant mice, increased in the *Robo2*^*PB*/+^ mutant mice, and significantly increased in the *Robo2*^*PB*/+^*Gen1*^*PB*/+^ mutant mice. The expression level of RET was increased in the *Robo2*^*PB*/+^, *Gen1*^*PB*/+^ and *Robo2*^*PB*/+^*Gen1*^*PB*/+^ mutant mice. According to Grieshammer et al. (18), the primary function of SLIT2/ROBO2 signaling during kidney development is to ensure that GDNF expression becomes localized to the region where the UB normally forms, thereby restricting kidney induction to the appropriate site. Therefore, we speculated that *Robo2* and *Gen1* may work together to regulate the GDNF/RET signaling pathway and play a synergistic role in promoting the expression of RET.

In general, the WD does not respond to the GDNF signal in anterior regions, and two potential explanations have been proposed (26). First, SLIT/ROBO signaling is known for its role in axon repulsion (21) and appears to repulse GDNF-expressing cells from the WD, resulting in a physical separation of these two structures in the anterior regions (19). Second, the anterior intermediate mesoderm (IM) exhibits relatively high levels of BMP signaling, which is thought to be an antagonist that suppresses ureter branching in kidney development (27). Heterozygous *Bmp4* mutations in mice lead to a variety of CAKUT phenotypes, including duplex kidneys (28).

In the *Robo2*^*PB*/+^*Gen1*^*PB*/+^ mutant mice, *Gen1* expression was reduced, accompanied by an increase in the expression of *Grem1*, consistent with previous studies (15). Previous studies found that both GREM1 and ROBO2 bind to the same domain of SLIT2 (29, 30). However, the affinity of ROBO2 for the N-terminal fragment of SLIT2 (SLIT2N) is much higher than that of GREM1 (31, 32). A high local concentration of GREM1 is needed to overcome the stronger interaction between SLIT2 and ROBO2. This finding has been verified in neurons, in which only high concentrations of GREM1 block SLIT2-mediated inhibition of neuronal migration. Therefore, GREM1 expression increased, which might result in competitive inhibition with ROBO2, reducing the binding between ROBO2 and SLIT2 (30). This effect may be exacerbated by the presence of a reduction in *Robo2* expression in the *Robo2*^*PB*/+^*Gen1*^*PB*/+^ mutant mice. GEN1 may interact with the ROBO2/SLIT2 signaling pathway through GREM1, thereby affecting the expression of GDNF/RET, but its specific mechanism remains to be further confirmed.

MM cells express the BMP inhibitor GREM1, which counteracts BMP function, to permit ureter outgrowth specifically at the proper site of the kidney (33). Both BMP2 and BMP4 are important members of the BMP signaling pathway. An *in vitro* kidney organogenesis experiment has shown that BMP2 treatment of cells causes a significant decrease in *GDNF* and *GFR*α*1* mRNA expression during differentiation from the posterior intermediate mesoderm stage to the metanephric mesenchyme (days 7–9) (30). In the *Robo2*^*PB*/+^*Gen1*^*PB*/+^ mutant mice, the increase in *Grem1* expression was accompanied by a decrease in BMP expression. Thus, we speculated that the increase in *Grem1* expression exerted an enhanced inhibitory effect on the BMP signaling pathway, which may lead to a decreased inhibitory effect of BMP2 and BMP4 on RET in the WD. Direct negative crosstalk between the SLIT2 and BMP-Gremlin signaling pathways has also been observed. A previous study revealed a negative feedback loop in fibroblasts in which SLIT2 inhibits GREM1 activity and BMP downregulates *Slit2* expression by suppressing *Slit2* promoter activity through canonical BMP signaling pathways (30). Through the negative crosstalk between these molecules, an interaction network was formed between *Robo2* and *Gen1*. We speculated that the combined actions of several molecules increase the expression of GDNF and RET.

**Figure 5:**
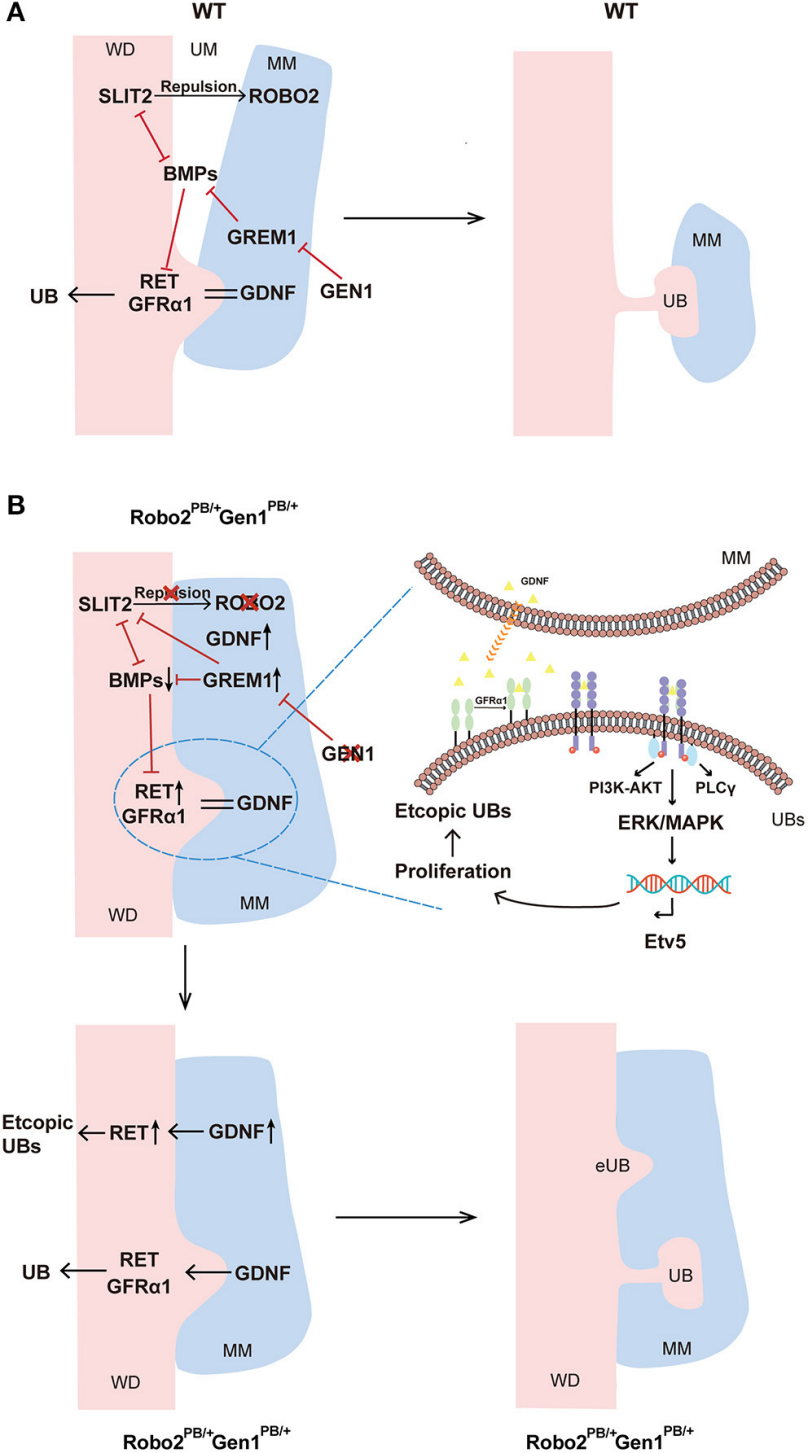
Molecular regulation of ureteric budding and branching morphogenesis. **(A)** In wild-type mice, GEN1 may inhibit GREM1 expression, and GREM1 counteracts BMP signaling. Additionally, direct negative cross-talk is observed between SLIT2 and BMP2-Gremlin signaling. BMP2, BMP4 and ROBO2 inhibit GDNF signal transduction, inhibiting budding and subsequent branching of the ureter via RET and GFRα1, thereby restricting kidney induction to the appropriate site. **(B)** In *Robo2*^*PB*/+^*Gen1*^*PB*/+^ mice, decreased GEN1 expression led to increased GREM1 expression, and the high local concentration of GREM1 competitively inhibited ROBO2, reducing the binding between ROBO2 and SLIT2. Prior to UB induction, the nephrogenic cord itself is expanded due to the reduced repulsion between ROBO2 and SLIT2 relative to wild-type. In contrast, the increase in *Grem1* expression exerted an enhanced inhibitory effect on the BMP signaling pathway, which may lead to the decreased inhibitory effect of BMP2 and BMP4 on RET in the WD. At the molecular level, RET is activated and recruited by GFRα1 and GDNF and subsequently activates downstream MAPK/ERK signaling and cell proliferation. As RET transcription factor, Etv5 reflects the activation of MAPK/ERK signaling, driving ectopic UB induction in more anterior metanephric/caudal mesonephric regions. *Gen1* and *Robo2* may exert a synergistic effect on activating the GDNF/RET pathway. WD, Wolffian duct; MM, metanephric mesenchyme; UM, ureteric mesenchyme; UB, ureteric bud; BMPs, BMP2 and BMP4.

As the major inducers of RET signaling in the early developing kidney, GDNF binding to its cognate receptors causes activation of intracellular signaling cascades, including the PI3K/AKT, RAS/MAPK and PLCγ/Ca^2+^ pathways (34). The MAPK/ERK pathway appears to be closely related to ureter branching, and mice lacking the MEK1 and MEK2 kinases do not form an appropriately branched ureteric tree (35). In the present study, we observed increased levels of pERK, which reflects MAPK signaling, in both the UB and its surrounding nephrogenic cord cells, while the levels of pAKT and pPLCγ were not changed in compound heterozygous mice. A previous study reported that Etv5 expression requires activation of the MEK/ERK (MAPK) signaling pathway in neurons (36). Significantly, in E12.5 *Robo2*^*PB*/+^*Gen1*^*PB*/+^ kidney sections, *Etv5* expression was increased in the UB tips. A previous study showed that MAPK/ERK activity regulates proliferation in UBs to achieve normal branching and growth (35). As a transcription factor, ETV5 is also dispensable for the cellular self-renewal ability (37). As the PHH3 occurs in late G2 and M phase of the cell cycle, it serves as a specific marker for cells undergoing mitosis (38). In this study, we detected the expression level of PHH3 and observed increased levels in double mutant mice. Therefore, we speculated that cell mitosis may be promoted through the activation of the downstream MAPK/ERK cascade, resulting in increased cell proliferation and finally the formation of ectopic UBs (Figures 5A,B).

In our study, pERK levels were increased in the UB and its surrounding nephrogenic cord cells in E11.5 *Robo2*^*PB*/+^*Gen1*^*PB*/+^ kidneys. In addition to regulating the UB epithelium, the MAPK/ERK pathway might also participate in the regulation of nephrogenesis, which has multiple functions in the guidance of nephron differentiation (34). NP-specific MAPK inactivation results in an almost complete lack of nephrons in newborn mouse pups (34, 39). Therefore, we speculate that ROBO2 and GEN1 not only coregulate ureteric budding but also exert some common effect on the nephrogenic cord through the activation of the cap mesenchyme marker SIX2 or MAPK/ERK signaling. Further studies are needed to address the combined effects of ROBO2 and GEN1 on nephron progenitor biology and the cap mesenchyme.

The specific interaction between *Gen1* and *Robo2* remains unclear. One interpretation of our results is that *Gen1* may interact with the ROBO2/SLIT2 pathway through *Grem1*. The different affinities of ROBO2 and GREM1 for SLIT2N may play a specific role in metanephric kidney development. The current data do not allow us to discriminate between these possibilities, which would require a detailed analysis of GREM1 and ROBO2. In addition, further studies will be required to search for mutation sites in the two genes in patients with CAKUT and to clarify their role in the development of the human kidney.

This finding is consistent with the recent understanding of CAKUT as an oligomeric/polygenic disorder that may be caused by the accumulation of multiple subtle mutations or polymorphisms (mutational load) that lead to dysfunction of the corresponding developmental program (40). Although some aspects of the roles of *Gen1* and *Robo2* in metanephros development remain to be clarified, our results unambiguously identified a cooperative role for *Gen1* and *Robo2* in metanephric budding.

## Data Availability Statement

The datasets presented in this study can be found in online repositories. The names of the repository/repositories and accession number(s) can be found in the article/Supplementary Material.

## Ethics Statement

The animal study was reviewed and approved by School of Life Sciences of Fudan University [Protocol Approval No. SYXK (hu) 2020-0011].

## Author Contributions

XW, HX, and QS conceived and designed research. YL performed experiments, analyzed data, and drafted the manuscript. MY, LT, SX, and XD interpreted results of experiments. YL, MY, and LT prepared figures. QS edited and revised the manuscript and approved final version of the manuscript. All authors contributed to the article and approved the submitted version.

## Funding

This work was supported by the National Natural Science Foundation of China (81670609 and 81900602) and the Establishment, Performance, and Quality Control of the Standardized Phenotype Analysis Process Grant (No. 2018YFA0801102).

## Conflict of Interest

The authors declare that the research was conducted in the absence of any commercial or financial relationships that could be construed as a potential conflict of interest.

## Publisher's Note

All claims expressed in this article are solely those of the authors and do not necessarily represent those of their affiliated organizations, or those of the publisher, the editors and the reviewers. Any product that may be evaluated in this article, or claim that may be made by its manufacturer, is not guaranteed or endorsed by the publisher.
