# Supplementary material for: Robo2 and Gen1 Coregulate Ureteric Budding by Activating the MAPK/ERK Signaling Pathway in Mice
Source: Front Med (Lausanne). 2022 Jan 5;8:807898. doi: 10.3389/fmed.2021.807898 (PMC8766746; doi:10.3389/fmed.2021.807898)
Supplement: Supplementary file 1 [file Table_1.DOCX]

You can access the raw data using the following link: <https://www.jianguoyun.com/p/De7skd0Qu53vCRj64ZUE>.

| **Figure number** | | **Contact** |
| --- | --- | --- |
| Figure 2 | Figure 2C | Images of the normal kidney at P0.5 |
|  | Figure 2D | Images of the duplex kidney at P0.5 |
|  | Figure 2E | Images of the hydronephrosis complicated with duplex kidney at P0.5 |
|  | Figure 2F-G | HE staining of on P0.5 kidneys sections |
| Figure 3 | Figure 3A | Normal ureteric budding at E11 |
|  | Figure 3B | Ectopic ureteric budding at E11 |
|  | Figure 3C | Normal ureteric budding at E11.5 |
|  | Figure 3D | Ectopic ureteric budding at E11.5 |
|  | Figure 3E | Normal ureteric budding at E12.5 |
|  | Figure 3F | Ectopic ureteric budding at E12.5 |
| Figure 4 | Figure 4A | IF staining with antibodies against pERK on E11.5 kidneys |
|  | Figure 4B | IF staining with antibodies against PHH3 on E11.5 kidneys |
| Figure S1 | Figure S1A | Images of the unilateral renal agenesis at P0.5 |
|  | Figure S1B | Images of the hydronephrosis at P0.5 |
|  | Figure S1C | Images of VUR in newborn mice P0.5 |
| Figure S2 | Figure S2A | IF staining with antibodies against pAKT on E11.5 kidneys |
|  | Figure S2B | IF staining with antibodies against pPLCγ on E11.5 kidneys |
| Figure S3 | Figure S4 | IF staining with antibodies against ETV5 on E12.5 kidneys sections |
