# Supplementary material for: Robo2 and Gen1 Coregulate Ureteric Budding by Activating the MAPK/ERK Signaling Pathway in Mice
Source: Front Med (Lausanne). 2022 Jan 5;8:807898. doi: 10.3389/fmed.2021.807898 (PMC8766746; doi:10.3389/fmed.2021.807898)
Supplement: Supplementary file 2 [file Table_2.DOCX]

Supplementary Material

# Materials and Methods

***VUR test***

Newborn mice were dissected to expose the kidneys and the urinary tract using an anterior midline incision. The bladder was punctured with a 25-gauge needle to manually inject methylene blue (1 mg/ml in PBS) at a rate of 100 µl/min. When the dye exited through the urethra, the transfusion device was closed, and a microscope was used to observe the presence of bladder ureter reflux.

***Immunofluorescence on Frozen Sections***

Fresh kidney tissue was fixed with 4% paraformaldehyde overnight, dehydrated in 30% sucrose for 48 h, embedded in optimal cutting temperature compound (OCT compound), and stored at -80℃. The sections were frozen and sliced at a thickness of 15 µm. The sections were washed with PBS, permeabilized with 0.3% Triton X-100 for 15 min, and blocked with 5% donkey serum for 1 h at room temperature. The tissue slices were incubated with a primary antibody at 4℃ overnight, washed several times with PBS, incubated with a secondary antibody at room temperature for 1 h, washed several times with PBS, and treated with an autofluorescence quencher. Slices were observed and imaged using a Zeiss-LSM880 with Airyscan confocal laser scanning microscope and ZEN ImageJ software (Zeiss, Germany). The following antibodies were used: primary antibodies: Rabbit anti-ETV5 (ProteinTech, 13011-1-AP, 1:100), secondary antibodies: Alexa 647-conjugated anti-rabbit (Jackson ImmunoResearch, 1:400). ImageJ was used to quantify the amount of fluorescence as mean gray value of Etv5 in the dashed circle with line and compare the results among four groups.

# Supplementary Figures and Tables

# 2.1 Supplementary Figures Legends

# Supplementary Figure 1. Analysis of newborn mouse phenotypes in the four groups at P0.5. (A-B) Representative images of unilateral renal agenesis (A) and hydronephrosis (B). (A#-B#) Representative images visualized using *Hoxb7* expression of unilateral renal agenesis (A#) and hydronephrosis (B#). (C-D) VUR in newborn mice. (C) Methylene blue refluxed to the renal pelvis, and no significant ureteral or renal pelvic dilatation was observed, but these phenotypes were never observed in the WT mice. (D) The percentage of VUR in the WT, *Robo2^PB/+^*, *Gen1^PB/+^* and *Robo2^PB/+^Gen1^PB/+^* newborn mice. Scale bars, 1 mm in (A-B#); 2 mm in (C). NS, not significant, P > 0.05; *, P < 0.05; **, P < 0.01.

**Supplementary Figure 2.** GDNF/RET signaling is enhanced in the *Robo2^PB/+^Gen1^PB/+^* mutants. **(A)** Immunofluorescence staining with antibodies against pAKT in E11.5 kidney sections shows similar pAKT signals in the WT, *Robo2^PB/+^*, *Gen1^PB/+^*and *Robo2^PB/+^Gen1^PB/+^* mice. N = 4, original magnification × 10. **(B)** Immunofluorescence staining with antibodies against pPLCγ in E11.5 kidney sections revealed similar pPLCγ signals in the WT, *Robo2^PB/+^*, *Gen1^PB/+^*and *Robo2^PB/+^Gen1^PB/+^* mice. N = 3 mice each in the *Gen1^PB/+^* and *Robo2^PB/+^* groups, N = 4 *Robo2^PB/+^Gen1^PB/+^* mice. Scale bars represent 100 μm in A-B.

**Supplementary Figure 3.** GDNF/RET signaling is enhanced in the *Robo2^PB/+^Gen1^PB/+^* mutants. Immunofluorescence staining with antibodies against Etv5 in the kidneys of the WT, *Robo2^PB/+^*, *Gen1^PB/+^*, *Robo2^PB/+^Gen1^PB/+^* mice at E12.5. The region circled by the dashed lines indicated the ureteric bud. N = 3, original magnification × 20. Scale bars represent 50 μm.

**Supplementary Table 1. (**The number of newborn mouse phenotypes in the four groups at P0.5.**)**

| **Phenotype** | **WT**  **N = 20** | **Robo2^PB/+^**  **N = 22** | **Gen1^PB/+^**  **N = 28** | **Robo2^PB/+^Gen1^PB/+^**  **N = 96** |
| --- | --- | --- | --- | --- |
| NK | 19 | 19 | 19 | 65 |
| DK | 1 | 1 | 7 | 29 |
| HN | 0 | 1 | 2 | 0 |
| URA | 0 | 1 | 0 | 0 |
| URA & DK | 0 | 0 | 0 | 2 |

NK: normal kidney; DK: duplex kidney; URA: unilateral renal agenesis; HN: hydronephrosis; WT: wildtypye.

Figure 1


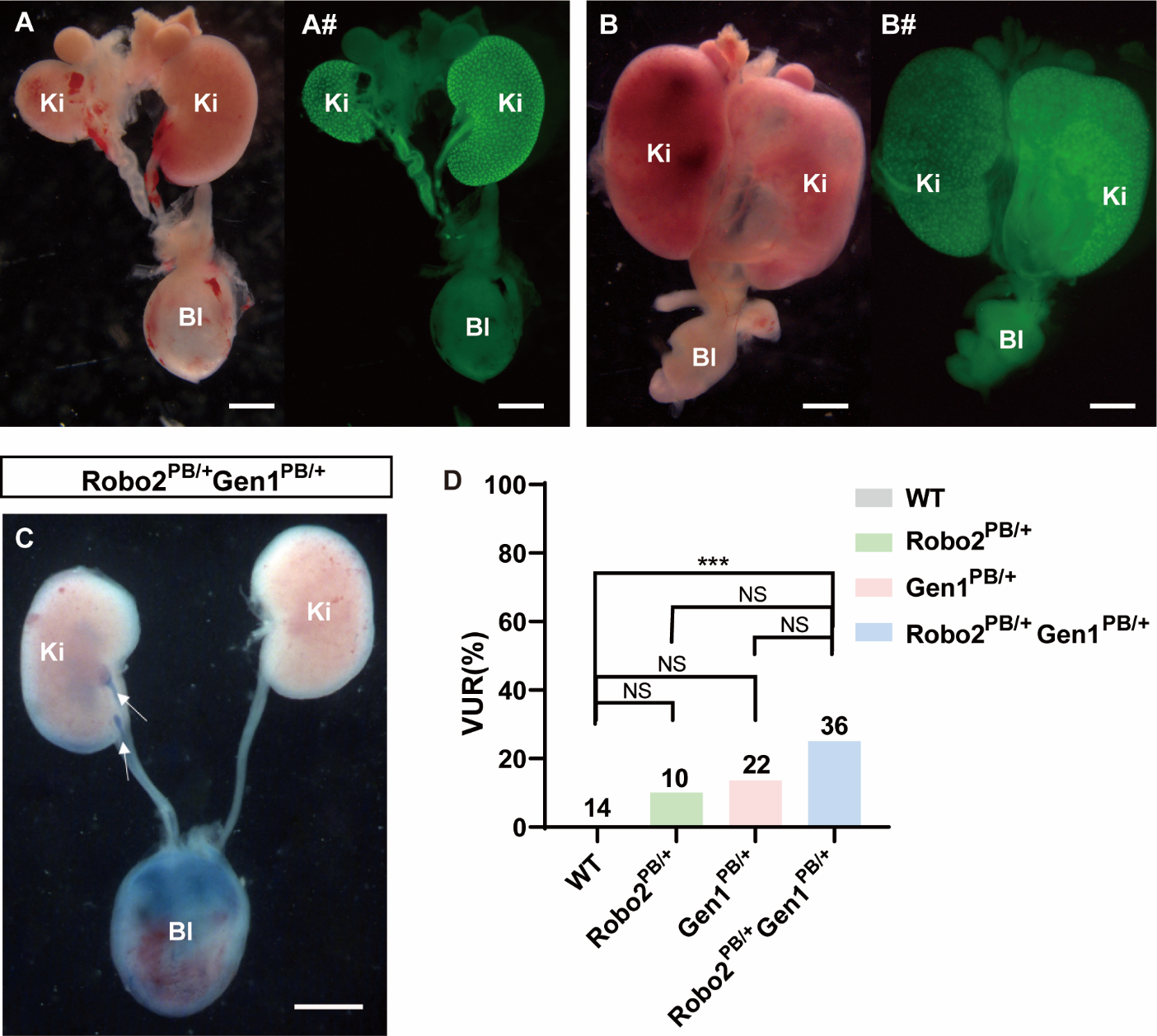


Figure 2


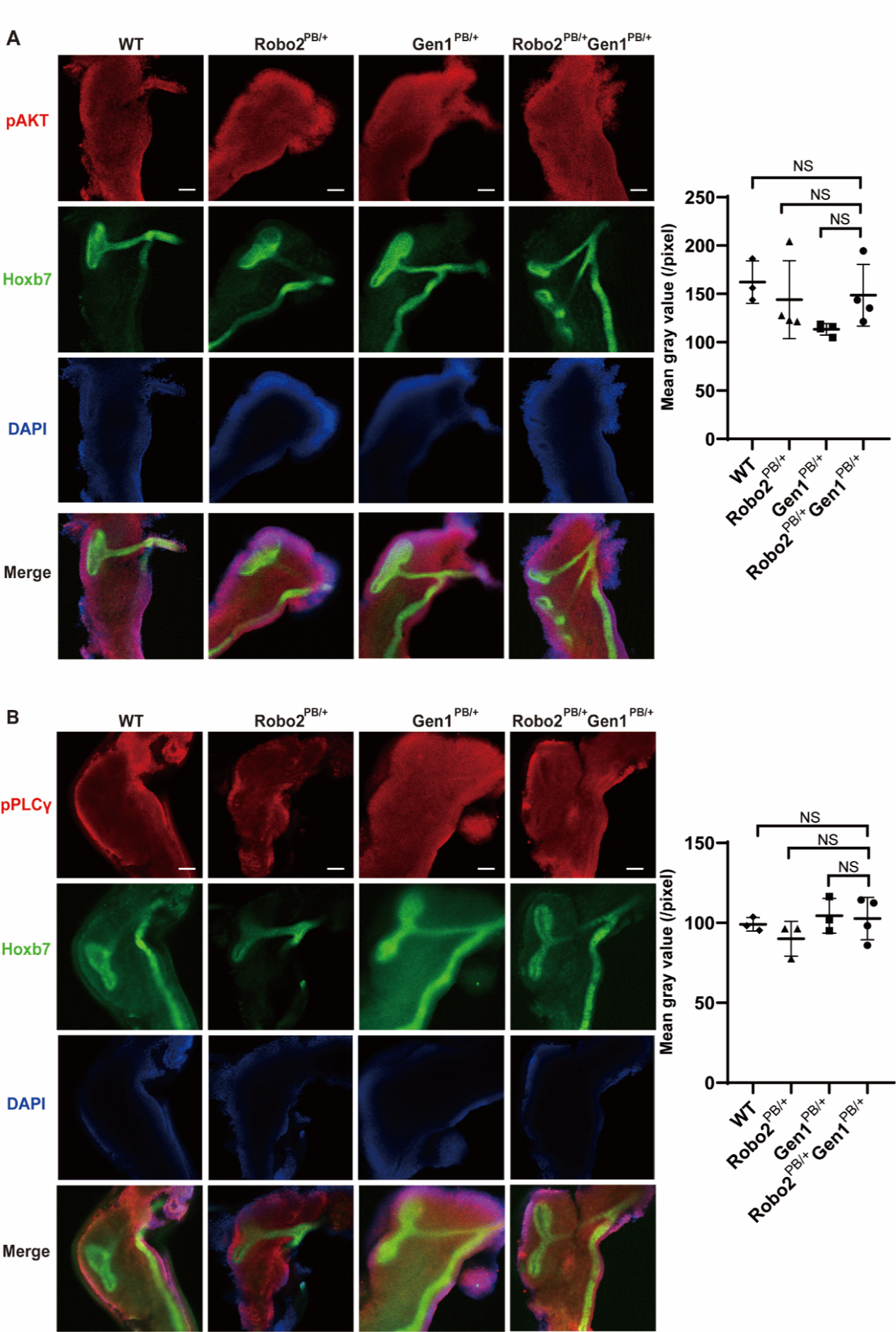


Figure 3


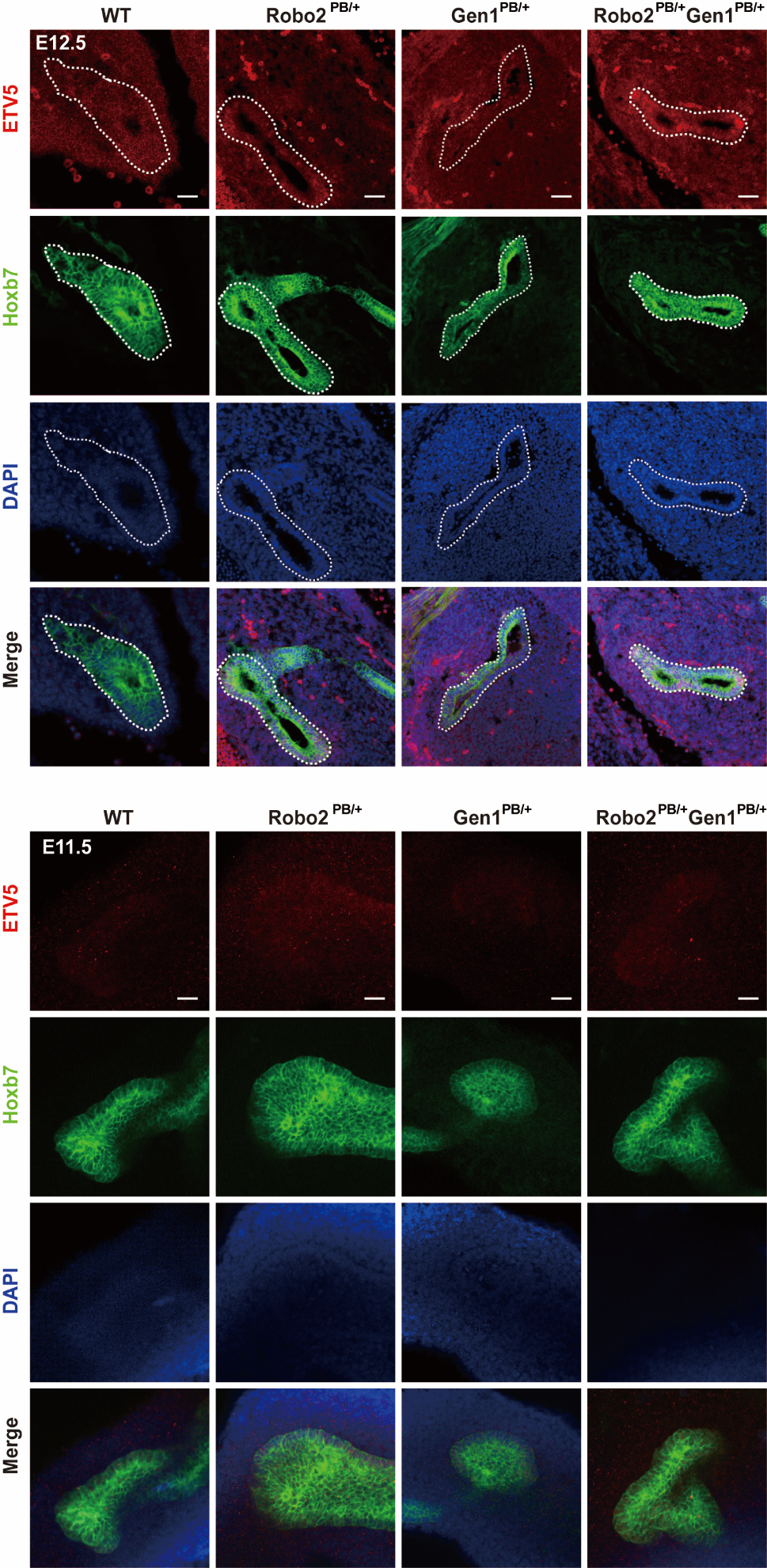


Supplemental Information can be found with this article online at DOI: 10.6084/m9.figshare.17170874
